# HIF-1α、COX-2和E-cadherin在肺腺癌的表达及临床意义

**DOI:** 10.3779/j.issn.1009-3419.2011.03.07

**Published:** 2011-03-20

**Authors:** 松涛 谷, 建文 秦

**Affiliations:** 1 300070 天津，天津医科大学研究生院 Graduate School, Tianjin Medical University, Tianjin 300070, China; 2 300051 天津，天津市胸科医院胸内科 Department of Thoracic Medicine, Tianjin Chest Hospital, Tianjin 300051, China

**Keywords:** 肺肿瘤, 低氧诱导因子-1α, 环氧合酶-2, 上皮型钙粘附分子, Lung neoplasms, Factor-1α, Cyclooxygenase-2, E-cadherin

## Abstract

**背景与目的:**

肺腺癌发病率不断升高，而低氧诱导因子-1α（hypoxia-inducible factor-1α, HIF-1α）、环氧合酶-2（cyclooxygenase-2, COX-2）、上皮型钙粘附分子（E-cadherin）均在肿瘤细胞的分化、增殖过程中起到重要作用。本研究旨在探讨HIF-1α和COX-2、E-cadherin在肺腺癌的表达水平与患者临床病理特征之间的关系及其三者之间的内在联系。

**方法:**

收集10例非肿瘤患者手术切除的正常肺组织及45例肺腺癌患者手术切除标本，应用免疫组织化学方法检测HIF-1α、COX-2、E-cadherin的表达情况。

**结果:**

45例肺腺癌组织中，HIF-1α和COX-2的表达阳性率分别为60%（27/45）和40%（18/45），10例正常肺组织均未见表达。45例肺腺癌组织中E-cadherin的表达阳性率为48.9%（22/45），10例正常肺组织均见阳性表达。HIF-1α表达水平与原发肿瘤大小有密切关系（*P* < 0.05），但与患者年龄、吸烟与否、淋巴结转移、分化程度、术后分期无明显关系（*P*>0.05）。COX-2表达水平与原发肿瘤大小、淋巴结转移、术后分期、HIF-1α表达水平有密切关系（*P* < 0.05），但与患者年龄、吸烟与否、分化程度无明显关系（*P*>0.05）。E-cadherin表达水平与分化程度、淋巴结转移有密切关系（*P* < 0.05），但与患者年龄、吸烟与否、肿瘤最大直径、术后分期、HIF-1α的表达无明显关系（*P*>0.05）。

**结论:**

肺腺癌组织中HIF-1α、COX-2表达增高，E-cadherin表达降低；COX-2的表达水平升高可能与HIF-1α的高表达有关，E-cadherin的表达水平与HIF-1α未发现有明显相关性。

## Abstract (en)

**Background and objective:**

The incidence of lung adenocarcinoma increases rapidly, and hypoxiainducible factor-1α (HIF-1α), cyclooxygenase-2 (COX-2), E-cadherin play an important role in the proliferation and differentiation of cancer cell. The aim of this study is to investigate the clinical significance of the expression of HIF-1α, COX-2, E-cadherin in patients with lung adenocarcinoma and the internal relationship among them.

**Methods:**

The expression levels of HIF-1α, COX-2 and E-cadherin were determined in 10 cases of normal lung issue and in 45 cases of issue of lung adenocarcinoma by immunohistochemical method.

**Results:**

The positive expression rate of HIF-1α and COX-2 was 60% (27/45) and 40% (18/45) respectively in 45 cases of lung adenocarcinoma, was null in 10 cases of normal lung issue. The positive expression rate of E-cadherin was 48.9% (22/45) in 45 cases of lung adenocarcinoma, was 100% in 10 cases of normal lung issue. The expression of HIF-1α was positively correlated with the size of the cancer (*P* < 0.05), but not with age, smoking, lymphatic metastasis, differentiated degree and surgical-pathologic staging (*P*>0.05). The expression of COX-2 was positively correlated with the size of the cancer, lymphatic metastasis, surgical-pathologic and expression of HIF-1α (*P* < 0.05), but not with age, smoking and differentiated degree (*P*>0.05). The expression of E-cadherin was positively correlated with differentiated degree and lymphatic metastasis (*P* < 0.05), but not with age, smoking, the size of the cancer, surgical-pathologic and expression of HIF-1α (*P*>0.05).

**Conclusion:**

The overexpression of HIF-1α and COX-2 and the downregulation of E-cadherin is found in lung adenocarcinoma. The overexpression of HIF-1α may induce the overexpression of COX-2, and is non-correlated with expression of E-cadherin.

实体肿瘤缺氧是一种普遍现象，肿瘤为适应低氧而发生一系列生物学改变，广泛存在于大多数实体肿瘤中的低氧诱导因子-1α（hypoxia-inducible factor-1α, HIF-1α）是这一系列改变的关键调控因子。肿瘤细胞通过高表达HIF-1α对其下游基因进行调控以适应低氧环境，促进肿瘤生长转移。有临床实验^[1]^证明环氧化酶-2在多种恶性肿瘤中有高表达。近来在胃癌的研究^[2]^中发现环氧化酶-2（cyclooxygenase-2, COX-2）受HIF-1α的调控，COX-2可能是HIF-1α下游目的基因，是肿瘤适应低氧的促进生存转移的重要途径，但尚缺乏肺腺癌的临床报道。上皮型钙粘附分子（E-cadherin）的缺失与肺腺癌侵袭转移有关，多项研究提示肺腺癌组织E-cadherin表达下降，同时在卵巢癌的研究中^[3]^发现HIF-1α和E-cadherin表达水平高度相关，而在肺腺癌中E-cadherin的表达是否也同时受HIF-1α的调控目前尚未见明确的报道。本研究联合检测三者在肺腺癌中的表达情况，探讨其与临床病理因素的关系及其可能存在的内在联系。

## 1 对象和方法

### 1.1 对象

45例肺腺癌来自天津市胸科医院2006年9月-2007年10月胸外科住院患者手术切除标本。经病理明确证实，且不伴有其它疾病，术前均未行放化疗。男性28例，女性17例，平均年龄62.8岁±6.8岁。临床分期根据1997年第八届国际肺癌大会公布的TNM分类标准，Ⅰ期20例，Ⅱ期10例，Ⅲ期15例。淋巴结转移阳性22例，阴性23例。高分化14例，中分化12例，低分化19例。正常对照组10例正常肺组织来自同期胸科医院非肿瘤患者手术切除的正常肺组织，且经病理证实。

### 1.2 方法

#### 1.2.1 主要试剂

HIF-1α鼠抗人单克隆抗体sc-53546，购于SANTA CRUZ公司。COX-2兔抗人单克隆抗体ZA-0515、E-cadherin鼠抗人单克隆抗体ZM-0092、二步法免疫组化检测试剂盒（PV-9000）、DAB显色试剂盒、APES防脱片胶工作液均购于北京中杉金桥试剂公司。

#### 1.2.2 主要仪器

石蜡切片机（德国Leica公司）、Olympus光学显微镜、医用微波炉。采用二步法免疫组化检测，步骤按试剂盒说明书操作。

### 1.3 结果判定

随机选择5个高倍视野，计数1, 000个肿瘤细胞，计算阳性细胞百分率，HIF-1α和COX-2阳性细胞百分率≥10%为阳性表达，E-cadherin阳性细胞百分率≤70%为表达下降，>70%为正常表达。HIF-1α蛋白染色阳性细胞标准为胞浆或胞核内可见棕黄色颗粒。COX-2蛋白染色阳性细胞标准为胞浆内出现棕黄色颗粒。E-cadherin蛋白染色阳性细胞标准为胞膜可见棕黄色颗粒。

### 1.4 统计学方法

采用SPSS 13.0软件包进行统计，免疫组化结果为计数资料采用*χ*^2^检验。*P* < 0.05有统计学差异。

## 2 结果

### 2.1 HIF-1α的蛋白表达与临床病理资料的关系

10例正常对照组均未见HIF-1α的表达。45例肺腺癌HIF-1α的阳性表达率为60%（27/45）。在肺腺癌组不同性别、年龄之间HIF-1α的表达无统计学差异（*χ*^2^=1.28, *P*>0.05; *χ*^2^=0.13, *P*>0.05）。HIF-1α的蛋白表达与患者是否吸烟无关（*χ*^2^=1.5, *P*>0.05）。淋巴结转移阳性组HIF-1α的阳性表达率（68.2%）与淋巴结转移阴性组（52.2%）相比差异无统计学意义（*χ*^2^=1.2, *P*>0.05）。HIF-1α阳性表达与肺腺癌的分化程度、术后分期无关（*χ*^2^=2.92, *P*>0.05; *χ*^2^=3.4, *P*>0.05）。肿瘤最大直径>2 cm组HIF-1α的阳性表达率（69.7%）明显高于≤2 cm组（33.3%），差异有统计学意义（*χ*^2^=4.85, *P* < 0.05）（表 1，图 1）。

**1:** HIF-1α的蛋白表达与临床病理资料的关系 Correlation between HIF-1α expression and clinicopathological factors in 45 patients with lung adenocarcinoma

Characteristic	*n*	HIF-1α		*χ*^2^	*P*
		(+)	(-)		
Gender				1.28	0.26
Male	28	15	13		
Female	17	12	5		
Age (year)				0.13	0.71
≥60	24	15	9		
< 60	21	12	9		
Smoking status				1.5	0.22
Yes	20	10	10		
No	25	17	8		
Tumor size				4.85	0.03
≤2 cm	12	4	8		
>2 cm	33	23	10		
Differentiation				2.92	0.23
Well	14	6	8		
Moderate	12	9	3		
Poor	19	12	7		
Lymphatic metastasis				1.2	0.28
(+)	22	15	7		
(-)	23	12	11		
Pathological stages				3.4	0.18
Ⅰ	20	9	11		
Ⅱ	10	7	3		
Ⅲ	15	11	4		

**1:**
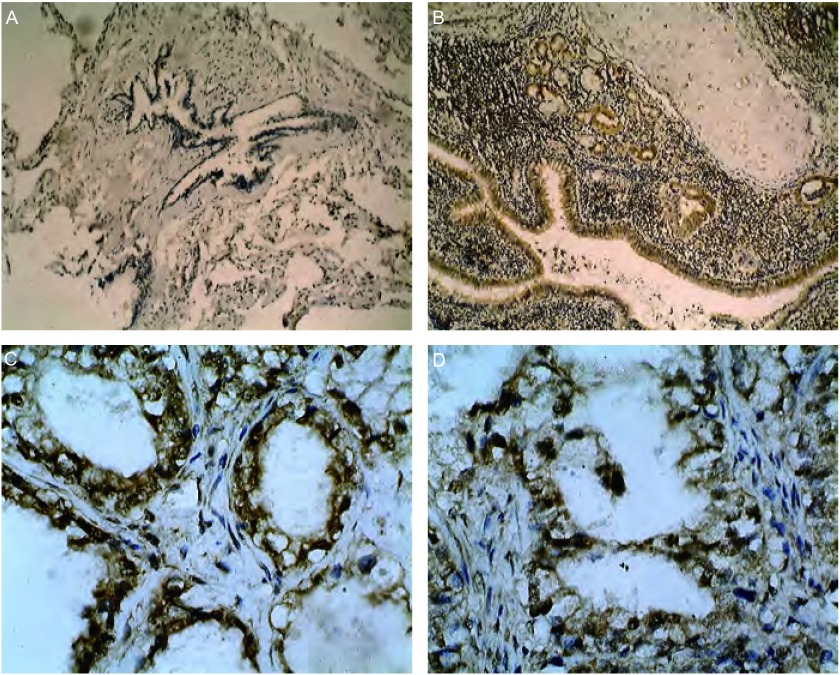
HIF-1α蛋白的表达。A：正常支气管粘膜上皮细胞HIF-1α蛋白表达阴性（SP, ×100）；B：肺腺癌切片相邻正常支气管粘膜上皮细胞HIF-1α阳性表达（SP, ×100）；C, D：HIF-1α在肺腺癌的阳性表达，腺癌细胞胞浆和胞核可见棕黄色颗粒（SP, ×400） Expression of HIF-1α Protein. A: negative expression in normal bronchial epithelium cells (SP, ×100); B: positive expression in normal bronchial epithelium cells next to the lung adenocarcinoma (SP, ×100); C, D: positive expression in the lung adenocarcinoma cells of which there are many brown particles in the cytoplasm and cytoblast (SP, ×400)

### 2.2 COX-2的蛋白表达与临床病理资料的关系

10例正常对照组均未见COX-2的表达。45例肺腺癌COX-2的阳性表达率为40%（18/45）。不同性别、年龄组之间COX-2的表达无差异（*χ*^2^=0.57, *P*>0.05; *χ*^2^=0.95, *P*>0.05）。COX-2的蛋白表达与患者是否吸烟无关（*χ*^2^=1.5, *P*>0.05）。COX-2表达在不同分化程度组之间无差异（*χ*^2^=0.16, *P*>0.05）。淋巴结转移阳性组COX-2的阳性表达率（63.7%）与淋巴结转移阴性组（17.3%）相比差异有统计学意义（*χ*^2^=10.0, *P* < 0.01）。肿瘤最大直径>2 cm组COX-2的阳性表达率（51.5%）明显高于≤2 cm组（8.3%），差异有统计学意义（*χ*^2^=6.84, *P* < 0.01）。COX-2阳性表达率在术后分期Ⅰ期组为15%明显低于Ⅱ期和Ⅲ期组（60%），具有明显的统计学差异（*χ*^2^=9.34, *P* < 0.01）。COX-2的表达与HIF-1α的表达有关，HIF-1α阳性表达组COX-2的阳性表达率（51.8%）明显高于HIF-1α表达阴性组（14.3%），差异具有统计学意义（*χ*^2^=3.95, *P* < 0.05）（表 2、表 3，图 2）。

**2:** COX-2的蛋白表达与临床病理资料的关系 Correlation between COX-2 expression and clinicopathological factors in 45 patients with lung adenocarcinoma

Characteristic	*n*	COX-2		*χ*^2^	*p*
		(+)	(-)		
Gender				0.57	0.45
Male	28	10	18		
Female	17	8	9		
Age (year)				0.95	0.33
≥60	24	8	16		
< 60	21	10	11		
Smoking status				1.5	0.22
Yes	20	6	14		
No	25	12	13		
Tumor size				6.84	0.009
≤2 cm	12	1	11		
>2 cm	33	17	16		
Differentiation				0.16	0.93
Well	14	5	9		
Moderate	12	5	7		
Poor	19	8	11		
Lymphatic metastasis				10.0	0.002
(+)	22	14	8		
(-)	23	4	19		
Pathological stages				9.34	0.009
Ⅰ	20	3	17			
Ⅱ	10	6	4		
Ⅲ	15	9	6		

**3:** HIF-1α表达与COX-2和E-cadherin表达的关系 Correlation among HIF-1α, COX-2 and E-cadherin

	HIF-1α		*χ*^2^	*P*
	(+)	(-)		
COX-2			3.95	0.047
(+)	14	4		
(-)	13	14		
E-cadherin			0.24	0.626
(+)	14	8		
(-)	13	10		

**2:**
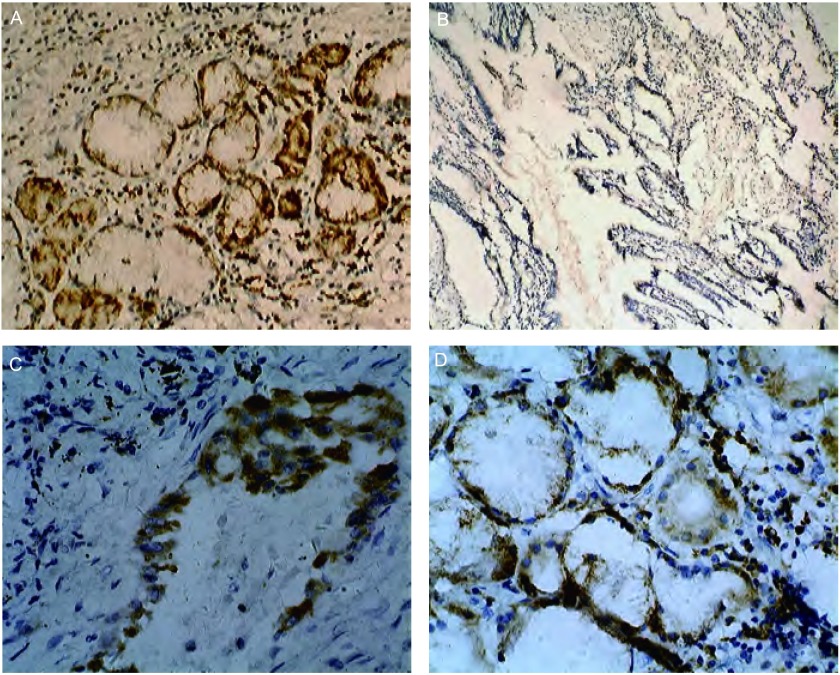
COX-2蛋白的表达。A：COX-2蛋白在肺腺癌的阳性表达，胞浆棕黄色的颗粒（SP，×100）；B：COX-2在正常支气管粘膜上皮细胞的阴性表达（SP，×100）；C, D：COX-2在肺腺癌阳性表达（SP，×400） Expression of COX-2 protein. A: positive expression in the lung adenocarcionoma of which there are many brown particles in the cytoplasm (SP, ×100); B: negative expression in normal bronchial epithelium cells (SP, ×100); C, D: positive expression in the lung adenocarcionma (SP, ×400)

### 2.3 E-cadherin的蛋白表达与临床病理资料的关系

10例正常对照组均见E-cadherin的阳性表达。45例肺腺癌E-cadherin的阳性表达率为48.9%（22/45）。不同性别、年龄组之间E-cadherin的表达无差异（*χ*^2^=1.08, *P*>0.05; *χ*^2^=1.84, *P*>0.05）。E-cadherin的蛋白表达与患者是否吸烟无关（*χ*^2^=2.78, *P*>0.05）。肿瘤最大直径>2 cm组E-cadherin的阳性表达率（45.4%）与≤2 cm组（60%）相比差异无统计学意义（*χ*^2^=2.07, *P*>0.05）。E-cadherin表达在不同分化程度组之间差异具有统计学意义（*χ*^2^=7.15, *P* < 0.05），随着分化程度下降表达率降低（71.4%, 58.3%, 26.3%）。淋巴结转移阳性组E-cadherin的阳性表达率（30.4%）与淋巴结转移阴性组（65.2%）之间的差异有统计学意义（*χ*^2^=5.02, *P* < 0.05）。E-cadherin阳性表达率在术后分期Ⅰ期组为60%，Ⅱ期组为50%，Ⅲ期组为33.3%，三者之间差异无统计学意义（*χ*^2^=2.45, *P*>0.05）。E-cadherin的表达与HIF-1α的表达无关（*χ*^2^=0.24, *P*>0.05）（表 3、表 4，图 3）。

**4:** E-cadherin的蛋白表达与临床病理资料的关系 Correlation between E-cadherin expression and clinicopathological factors in 45 patients with lung adenocarcinoma

Characteristic	*n*	E-cadherin		*χ*^2^	*P*
		(+)	(-)		
Gender				1.08	0.30
Male	28	12	16		
Female	17	10	7		
Age (year)				1.84	0.18
≥60	24	14	10		
< 60	21	13	8		
Smoking status				2.78	0.09
Yes	20	7	13		
No	25	15	10		
Tumor size				2.07	0.15
≤2 cm	12	8	4		
>2 cm	33	14	19		
Differentiation				7.15	0.03
Well	14	10	4		
Moderate	12	7	5		
Poor	19	5	14		
Lymphatic metastasis				5.02	0.03
(+)	22	7	15		
(-)	23	15	8		
Pathological stages				2.45	0.29
Ⅰ	20	12	8		
Ⅱ	10	5	5		
Ⅲ	15	5	10		

**3:**
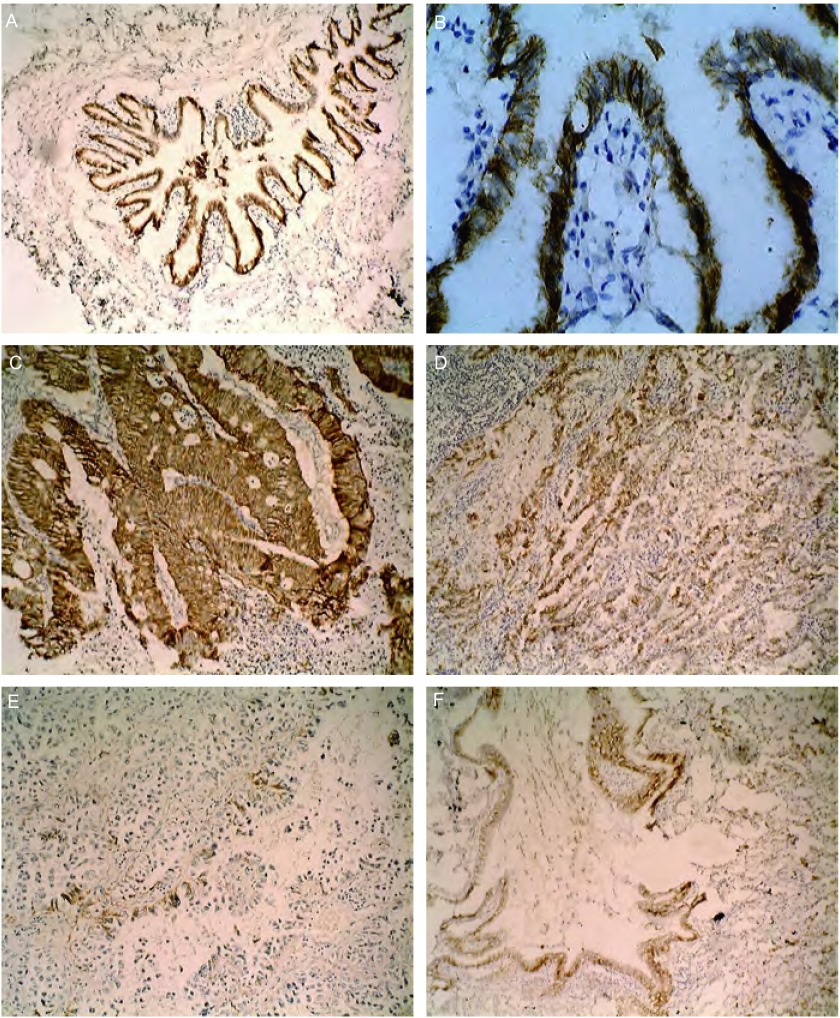
E-cadherin蛋白的表达。A：E-cadherin在正常支气管粘膜上皮细胞的正常表达，胞膜可见棕色颗粒沉着（SP，×100）；B：E-cadherin在正常支气管粘膜上皮细胞的正常表达（SP，×400）；C：E-cadherin蛋白在高分化腺癌的表达（SP，×100）；D：E-cadherin蛋白在中分化腺癌的表达（SP，×100）；E：E-cadherin蛋白在低分化腺癌的表达（SP，×100）；F：E-cadherin蛋白在肺腺癌相邻正常支气管粘膜上皮的表达（SP，×100） Expression of E-cadherin protein. A: positive expression in normal bronchial epithelium cells of which there are many brown par ticles in the cell membrance (SP, ×100); B : positive staining in normal bronchial epithelium cells (SP, ×40 0); C: expression in the lung welldif ferentiated adenocarcinoma (SP, ×100); D: expression in the lung moderate-differentiated adenocarcinoma (SP, ×100); E: expression in the lung poor-differentiated adenocarcinoma (SP, ×100); F: expression in normal bronchial epithelium cells next to the lung adenocarcinoma (SP, ×100)

## 3 讨论

HIF-1α最早是1992年由Semenza和Wang在低氧的肝细胞癌株Hep3B细胞核提取物中发现的一种核转录因子，其由HIF-1α和HIF-1β两个亚基单位组成，其转录活性主要由HIF-1α的表达水平和稳定性决定^[4]^。它调控着下游众多基因如血管内皮生长因子、胰岛素样生长因子、内皮素-1，促红细胞生成素等的转录和表达，是低氧诱导基因转录信息传递的最主要途径^[5]^。低氧是实体瘤发展过程中的普遍现象，而HIF-1α是关键调控因子。HIF-1α蛋白表达受细胞内氧浓度的精确调控。大量的体外实验显示，在5%的氧含量（50 mmHg）时HIF-1α被激活，随着氧浓度减少到0.1%-0.2%，其活性逐渐增加，在无氧时消失。肿瘤细胞通过高表达HIF-1α对其下游基因进行调控以适应低氧环境，促进肿瘤生长转移^[6]^。一般情况下，HIF-1α在正常组织及良性病变组织中无表达或仅有弱阳性表达，而在一些癌前病变组织、癌组织中过量表达^[7]^。本研究得到了相似的结论，肺腺癌组HIF-1α的阳性表达率明显高于对照组，同时我们也发现随着肿瘤直径的增大HIF-1α的表达阳性率相应增高，提示细胞缺氧发生在癌前病变之前，持续肿瘤发生的全过程，而HIF-1α异常表达可能是癌变过程的早期行为。

环氧合酶是花生四烯酸合成前列腺素的限速酶，是一种膜结合蛋白，有COX-1和COX-2两种同工酶。COX-1在大多数组织中持续低浓度表达，参与机体生理功能的调节；而COX-2是一种诱导型酶，除部分中枢神经系统及肾脏组织外在正常组织中通常不表达，但在某些病理条件下表达上调。近年来，流行病学、动物实验、临床试验等多方面的证据表明，COX-2与肿瘤的发生和发展有关^[8]^。COX-2可以通过促进细胞异常增殖、抑制细胞凋亡等机制参与调控肿瘤生长、浸润转移及血管形成等诸多病理过程。本研究显示，与非肿瘤患者的正常肺组织相比，肺腺癌组织中COX-2的表达阳性率明显升高，COX-2阳性表达与患者肿瘤的大小、淋巴结转移情况和临床分期有关，提示COX-2参与了肺腺癌的发生、浸润和转移，是肺腺癌发生的一个早期事件和预后不良的指标，其促进肺腺癌发生和发展的机制可能为：①促进肿瘤细胞增殖；②抑制细胞凋亡；③促进肿瘤淋巴管的形成^[9]^；④刺激血管因子产生，促进血管内皮细胞游走和血管形成^[10, 11]^；⑤参与致癌物的代谢^[12]^；⑥增强肿瘤细胞的侵袭力^[13]^。

E-cadherin是一类介导细胞与细胞间互相粘附的钙依赖性跨膜糖蛋白，它广泛存在于上皮细胞中，通过胞浆连环素与细胞骨架蛋白连接，以维护上皮细胞的形态和结构的完整性^[14]^。E-cadherin表达异常是导致细胞间粘附下降的重要原因。而肿瘤细胞间的粘附下降是导致肿瘤发生、发展的重要原因之一。体外细胞培养提示当肿瘤细胞E-cadherin表达下调或功能障碍时，可导致肿瘤细胞具有侵袭性生长的特点，并容易脱离原发部位向局部淋巴结或远处转移^[15, 16]^。目前有关E-cadherin与肺腺癌发生、侵袭转移及预后的关系报道极少。本研究显示E-cadherin异常表达与肺癌区域淋巴结转移有关，有区域淋巴结转移的肿瘤组织异常表达率明显高于无转移者，并且发现E-cadherin表达与肺腺癌细胞的分化程度有关，随着分化程度下降表达率降低。以上说明E-cadherin不仅与肺腺癌区域淋巴结转移有关，是判断肺腺癌淋巴结转移的一个重要指标，而且与肺腺癌的恶性程度有关。因此E-cadherin可能是临床判断肺腺癌淋巴结转移、癌细胞分化程度及评估预后的一个较好指标。

有学者^[17]^在肝癌和非小细胞肺癌的研究中发现，COX-2和HIF-1α表达呈正相关性。Csiki等^[18]^研究发现在A549等多种肺癌细胞株中，HIF-1α可以通过直接结合COX-2启动子上的HRE反应元件上调COX-2的表达，推测两者在癌细胞的发生、发展过程中具有协同作用。本研究得出了相似的结果，HIF-1α阳性表达组COX-2的阳性表达率明显高于HIF-1α表达阴性组，提示COX-2的表达与HIF-1α的表达有关。由此可见COX-2可能是HIF-1α的下游目的基因，受HIF-1α的调控。众所周知，恶性肿瘤细胞增殖异常迅速，不可避免地造成了低氧的微环境，此时作为急性缺氧的分子标志HIF-1α表达相应增高并同时诱导下游*COX*-2基因的表达，并可能通过这一下游路径诱导血管生成，改善低氧的微环境，从而促进肿瘤的发生、侵袭与转移。而较早提出HIF-1α与E-cadherin之间的关联是在卵巢癌^[3]^中：免疫组化提示核染色的HIF-1α与E-cadherin的缺失高度相关，且体外实验提示了其内在关联，但通过本研究，我们未能得到相似的结论，研究结果显示肺腺癌组织中E-cadherin与HIF-1α的表达无统计学意义。分析原因可能与样本含量相对较少有关，这还有待于做进一步更大样本的深入研究分析。

总之，恶性肿瘤的形成和转移是一个多基因、多因子共同作用的结果。通过这个研究提示我们，HIF-1α可能通过调控COX-2的表达，对肿瘤的生长转移起促进作用；虽然未发现E-cadherin和HIF-1α的相关性，尚需更大样本的研究，但有一点可以肯定，HIF-1α、COX-2和E-cadherin均参与了肺腺癌细胞的发生、浸润和转移。联合检测HIF-1α、COX-2和E-cadherin将有助于临床评估病情和判断预后。而且近年来，肺腺癌的靶向治疗已经作为研究的热点，有些靶向治疗药物已在临床上得到了应用并收到了很好的治疗效果。随着研究的深入，HIF- 1α、COX-2和E-cadherin相互关系的进一步阐明，多靶点治疗可能会为肺腺癌综合治疗提供一个新的武器，从而有助于抑制肿瘤细胞增殖，减少复发和转移，改善患者的生活质量。
